# Comparing the effectiveness of clomipramine and fluoxetine in dogs with anxiety-related behaviours

**DOI:** 10.18849/ve.v9i1.679

**Published:** 2024-03-13

**Authors:** Olivia Williamson+, Valery Varela+, Juliana Tom+, Elizabeth Powell+, Christopher Minami+, Jeffrey Norris+

**Keywords:** ACRAL LICK DERMATITIS, ANXIETY, ANXIOLYTIC, BEHAVIOURAL DISORDER, CANINE, CLOMIPRAMINE, DOG, FLUOXETINE, OBSESSIVE COMPULSIVE BEHAVIOUR, STEREOTYPIC BEHAVIOUR, TAIL CHASING

## Abstract

**PICO question:**

In dogs with anxious behaviours, is fluoxetine more effective than clomipramine in reducing anxiety-related behaviours?

**Clinical bottom line:**

**Category of research:**

Treatment.

**Number and type of study designs reviewed:**

Three controlled studies were critically appraised.

**Strength of evidence:**

Moderate.

**Outcomes reported:**

Administration of either fluoxetine or clomipramine to adult dogs reduces symptoms of fear and anxiety.

**Conclusion:**

Both fluoxetine and clomipramine are effective in reducing acral lick dermatitis and tail chasing behaviours, but there is no evidence that one drug is more effective than the other.

## How to apply this evidence in practice

The application of evidence into practice should take into account multiple factors, not limited to: individual clinical expertise, patient’s circumstances and owners’ values, country, location or clinic where you work, the individual case in front of you, the availability of therapies and resources.

Knowledge Summaries are a resource to help reinforce or inform decision-making. They do not override the responsibility or judgement of the practitioner to do what is best for the animal in their care.

## Clinical scenario

An owner brings a dog into a clinic that presents with an anxiety-related, repetitive behaviour, such as acral lick dermatitis or tail chasing. Allergies, parasitism, and infection have been ruled out. The owner has not found behavioural training for the dog to be effective and is wondering if there is a medication that can help reduce such repetitive behaviours. The veterinarian is aware of two drugs that are effective treatment options: fluoxetine and clomipramine. They both take 4–8 weeks to have a therapeutic effect. Knowing it may take months to alleviate this patient’s symptoms, the veterinarian wants to choose the more effective drug first.

## The evidence

Between the three identified studies, the evidence is not strong enough to choose one drug over the other for the treatment of anxiety-related, repetitive behaviours in dogs. Both clomipramine and fluoxetine are effective in decreasing behaviours such as tail chasing or acral lick dermatitis (Rapoport et al*., *1992; Yalcin, 2010; and Unnithan et al*., *2021). The identified studies were based on trials with low numbers of participants over relatively short periods of time. Rapoport (1992) reported on long-term outcomes for the participants, and these outcomes were limited to qualitative accounts by owners. 

The evidence includes three randomised controlled trials, Rapoport (1992) and Yalcin (2010) were double-blinded and one that did not claim to be blinded. Yalcin (2010) did not find a significant difference between the use of clomipramine and fluoxetine in treatment of tail chasing. However, both medications were generally effective in reducing this behaviour. This manuscript is limited by the small  sample population 25 dogs), which is skewed young (median participant was 36 months old), and in which some breeds were over-represented. Rapoport et al. (1992) was a slightly larger study (37 dogs) and included a crossover study design. However, the study design did not statistically compare fluoxetine to clomipramine directly, but both showed a similar decrease in licking behaviour. Unnithan et al. (2021) treated dogs that presented with chronic acral lick dermatitis with either fluoxetine or clomipramine. Although the dogs had improved symptoms, there was not a clear statistical significance when given one medication versus the other. Additionally, blinding was not reported as part of the study design, which raises the possibility of bias that may confound the results. Other limitations of the studies are presented below and should be considered when choosing either medication for long- term treatment of these behaviours.

All of the reports found both drugs to be effective but were limited by short time frames and small numbers of participants. Based on the data presented in these articles, there is not sufficient evidence to discriminate between the use of either fluoxetine or clomipramine in the treatment of anxious behaviours in dogs. More research should be directed towards better defining adverse effects and determination of long-term outcomes with treatment.

## Summary of the evidence

**Rapoport et. al (1992):** 

Population	Dogs of different ages, breeds, and sexes with a minimum 6-month history of chronic acral licking that produced lesions and had been unresponsive to treatment.
Sample Size	37 dogs.
Intervention Details	Three 11-week crossover trials were conducted for different pairs of drugs. There were two 5 week long arms of the study with a wash out period in between. For each comparison, Group 1 was treated with one drug for 5 weeks while Group 2 was treated with a second drug. The drugs were switched between the two groups for weeks 6–10: Study 1 (n = 13): clomipramine vs desipramine hydrochloride. Study 2 (n = 14): fluoxetine hydrochloride vs fenfluramine hydrochloride. Study 3 (n = 10): sertraline hydrochloride vs placebo. However, this does not relate to the PICO question and therefore will not be commented on further in this Knowledge Summary. For clomipramine, desipramine hydrochloride, and sertraline hydrochloride, doses were increased up to 3 mg/kg per day, as tolerated by the dog. Starting doses were not reported. For fluoxetine hydrochloride and fenfluramine hydrochloride, doses were increased up to 1 mg/kg per day, or as tolerated by the dog. Starting doses were not reported. The owners were not told which treatment their dog was receiving but were aware that two drugs were being compared. The authors report that the study was double-blinded but did not provide more details. Owner was interviewed by telephone each week of the 11 week trial as to licking behaviour and any side effects: Licking was scored on a 10-point scale (0 being an absence of licking). A baseline licking score was taken as a control 1 week before the study began.
Study design	Randomised, controlled double-blinded trial.
Outcome studied	Licking behaviour for different drug treatments, as reported by the owner. Adverse effects, as reported by the owner.
Main findings (relevant to PICO question)	Decrease in acral licking for clomipramine (average 43% decrease from baseline, P < 0.05): 6/13 subjects had 50% or more reduction of licking behaviours. The average reduction was not reported. Decrease in acral licking for fluoxetine (average 39% decrease from baseline, P < 0.05): 4/14 subjects had 50% or more reduction of licking behaviours. The average reduction was not reported. 2/14 subjects showed full remission of symptoms. Greatest decrease in acral licking across all treatments was observed in the fluoxetine treatment by week 4–5. Clomipramine and fluoxetine were both significantly more effective than the other treatments, but no significant difference was observed between fluoxetine and clomipramine. Adverse effects: Adverse effects were ‘mild’ and ‘often subsided over time’. Clomipramine – 5/13 subjects experienced at least one side effect: Lethargy (n = 3), loss of appetite (n = 2), diarrhoea (n = 1), and growling (n = 1). Fluoxetine – 4/14 subjects experienced: Lethargy (n = 2), loss of appetite (n = 1), hyperactivity (n = 1). 6-month follow-up: Clomipramine – of the 6/13 subjects that experienced 50% or more reduction of licking behaviours, only 2 continued treatment. Fluoxetine – of the 6/14 subjects that experienced 50% or more reduction of licking behaviours or complete remission: two continued improvement without medication one continued treatment with good response one discontinued treatment due to lack of further response two were euthanised for ‘nonrelated medical problems’, both of which were cancerous.
Limitations	Standard deviation was not reported with their data comparing mean response to all treatments. Initial dose given to subjects was not provided. Method for increasing dose was not provided. Not all subjects received the same final dosage of the drug. Only results for the first arm of the crossover were reported for each treatment Duration of washout period was not explicitly stated. No results reported regarding the comparison of drugs between the first and second arms for individual subjects. No rationale given for the different dosages used in each crossover study. Food allergy was not ruled out as a possible cause of acral dermatitis prior to treatment. Random allocation sequence not explained: Distribution of subjects for each treatment not stated. Possible misrepresentation of results depending on distribution of subjects (i.e. if all intact males were overrepresented in a certain treatment). Results based on subjective owner reports. Limited number of subjects: Only a few breeds are represented in the study; 13 subjects are listed as ‘other’. Intact females are under-represented in the sample size.

**Yalcin (2010):** 

Population	Dogs of different age, sex, and breed with tail chasing episodes for a minimum of 60 sec per episode.
Sample Size	25 dogs.
Intervention Details	Eight dogs given clomipramine hydrochloride 2 mg/kg BID 10 minutes before feeding for 12 weeks. Nine dogs given fluoxetine hydrochloride 1 mg/kg SID 10 minutes before feeding for 12 weeks. Eight dogs given placebo, 2.5 mg dextrose administered in gelatin capsules, SID 10 minutes before feeding for 12 weeks. Each subject received 30 minutes of exercise daily during the 12-week treatment period and was fed a low-protein diet (16–20% protein on a dry matter basis).
Study design	Randomised, controlled double-blinded trial.
Outcome studied	The response to treatment with clomipramine and fluoxetine in dogs with tail chasing was measured using score sheets: Owners assigned a score based on a scale: 0 = no change observed, 1 = minimal improvement, 2 = moderate improvement, to 4 = substantial improvement. Owners of dogs from each treatment group were contacted weekly to provide information: on hours spent with the dog per day number of tail chasing episodes per day duration of the longest episode whether the episode ended spontaneously or by interruption whether the owner had observed any adverse drug effects. The responses were evaluated in four intervals: weeks 1–3, weeks 4–6, weeks 7–9, and weeks 10–12.
Main findings (relevant to PICO question)	Clomipramine and fluoxetine were more effective in reducing signs of tail chasing than the placebo: Clomipramine reduced tail chasing behaviour more significantly than the placebo in all intervals (P < 0.05). Compared to the placebo group, the fluoxetine group improved significantly between weeks 7–9 and 10–12 (P < 0.05). Clomipramine and fluoxetine were not significantly different during any treatment interval (P > 0.05). Adverse effects or worsening of behaviour in dogs treated with clomipramine or fluoxetine were not noted.
Limitations	German Shepherd dogs and Anatolian Sheepdogs were over-represented. The data is insufficient to evaluate gender effects because only 1/8 dogs in the clomipramine group and 2/9 dogs in the fluoxetine group were female. Does not specify how randomisation and double-blinding was achieved. Population size was small and not equal for each treatment group. Results were subjective owner reports. 10/25 dogs had their tails bandaged during the study and it was not noted if these dogs received any additional interventions (e.g. medications administered). Home environments may have varied which may have affected behavioural outcomes. Not all subjects may have received the 30 minutes of daily exercise, been fed the low-protein diet, or had the drugs administered reliably. No follow-up contact with owners occurred after the 12 week treatment periods to determine long-term outcomes for dogs, such as the durability of response or continued use of medication by owners.

**Unnithan et. al (2021):** 

Population	Dogs of unspecified age and sex from the Small Animal Dermatology Unit of the Madras Veterinary College Teaching Hospital with visible alopecia and ulcerations resulting from excessive licking of their limbs.
Sample Size	16 dogs.
Intervention Details	All dogs received antibiotics for 10 days based on antibiogram results. Group I: eight dogs received clomipramine 2 mg/kg twice daily for 6 weeks. Group II: eight dogs received fluoxetine 1 mg/kg once daily for 6 weeks.
Study design	Randomised, controlled trial.
Outcome studied	Beneficial changes in licking behaviour were documented after 4 and 6 weeks as reported through a Likert scale with treatment of an anxiolytic medication.
Main findings (relevant to PICO question)	Average decrease in licking after treatment with clomipramine: After Week 4: 10%. After Week 6: 30%. Average decrease in licking after treatment with fluoxetine: After Week 4: 30%. After Week 6: 40%.
Limitations	Different antibiotics were potentially given to each subject prior to the start of testing, as antibiotic was chosen based on individual antibiogram results. Small population size. Breeds, ages, and sexes of subjects were unspecified. Authors did not specify that the study was blinded. Questions scored using Likert scales were not provided, and results of scoring were not provided. Results of Whitney U test were not provided. No control groups, such as a placebo group, were used for comparison. Since the dermatitis was marked and required treatment with antibiotics, data concerning wound healing over the course of the study might have provided additional evidence as to the efficacy of the treatment. Food allergy was not ruled out as a possible cause of acral dermatitis.

## Appraisal, application and reflection

Three studies were identified that answer the PICO question. Each study looked at both fluoxetine and clomipramine; Yalcin (2010) and Rapoport et al. (1992) both compared to a placebo control but Unnithan et al. (2021) did not compare to a control. Each study used randomisation to determine treatment groups. All three of these studies include bias due to reliance on scoring done by pet owners. There were no articles published with higher levels of evidence.

Yalcin (2010) found that both fluoxetine and clomipramine were equally effective at reducing tail chasing. Specifically, a significant difference was reported between clomipramine and placebo, and no difference between fluoxetine and clomipramine, but it was not reported whether fluoxetine differed significantly from the placebo. During the final interval (weeks 10–12) of the study, 7/8 dogs in the clomipramine group, 8/9 dogs in the fluoxetine group, and 1/8 dogs in the placebo group had moderate to substantial improvement. A statistical analysis of these results was not provided. As improved scoring improved in all three groups, including the control, age or other factors may contribute to the results.

Rapoport et al. (1992) was a blinded study and used a crossover design, though they did not report on the results of the second phase of the study. The wash out period between the different treatment phases was not directly reported for the study, but was inferred to be 1 week.  Fluoxetine and its metabolite would have required a 10 day wash out period, based on the five elimination half-lives of the drug. In this study clomipramine was compared to desipramine as a control, and fluoxetine to fenfluramine. In comparing all the drugs in the study clomipramine and fluoxetine were found to be equally effective.

Unnithan et al. (2021) did not specify any type of blinding in their study. An unspecified number of the dogs in this study were treated with antibiotics for epidermal ulceration associated with licking, though the authors did not state the antibiotics used or the degree to which injuries healed and, instead, reported on changes in licking behaviour as scored by owners. A stronger effect was reported for the fluoxetine treatment group compared to the clomipramine group, though no statistical analysis of the results was provided. Neither Unnithan et al. (2021) or Rapoport et al. (1992) addressed allergy testing or treatment despite environmental and food allergies being possible causes for acral lick granulomas.

Dogs can manifest anxiety through a variety of activities including tail chasing and licking. In the longer-term follow-up reported by Rapoport et al. (1992) three of the patients continued treatment with favourable results for at least 6 months after the study, one with fluoxetine and two with clomipramine. This may indicate some efficacy for longer-term management of acral lick dermatitis. There may also be success in resolving symptoms, as it was also reported that two of the patients on fluoxetine continued improvement following discontinuation of the drug. However, individual responses may vary, because in one instance fluoxetine treatment was discontinued due to no further improvement.

Licking is a behaviour that dogs can exhibit compulsively that can lead to self-injury in the form of acral lick dermatitis, however, acral lick dermatitis is also reported to be caused by environmental and food allergies. Neither Rapoport et al. (1992) or Unnithan et al. (2021) addressed allergies as a potential cause of acral lick granulomas. Had other causes for acral lick dermatitis been included during subject enrollment, those candidates could have been rejected for the study and a higher number of patients may have responded to the drugs*.* Even without addressing allergies, improvement in treatment groups was reported in both studies. This information could be helpful in the treatment of animals suffering from acral lick dermatitis whose owners are not able to pursue allergy testing or food trials.

Though there is a limited amount of research available to compare clomipramine and fluoxetine in treatment of anxious behaviour, there is evidence that they are both effective treatment options. Since other causes of acral lick dermatitis were not eliminated, a greater proportion of patients could have responded to treatment. Other possible considerations when choosing which of these drugs to prescribe include cost to the owner and durability of response. It is also possible that dogs may have a more robust response to one drug or the other on an individual basis and trial-and-error may be required to find the best treatment. Additional research should be done to compare these two commonly used drugs to strengthen the evidence that currently suggests that the two are similarly effective, along with addressing some of the shortcomings of these trials.

## Methodology

**Search strategy:** 

Search terms	CAB Abstracts via VetMed Resource (2010–2023) PubMed via National Institutes of Health (1990–2023)
Dates searches performed:	CAB Abstracts: (("clomipramine" OR "Anafranil" or "clomicalm") AND ("fluoxetine" OR "Prozac" OR "Sarafem") AND ("Anxiety" OR "behavior" OR "behaviour" OR "anxious" OR "fearful" OR "fear" OR "stress" OR "licking" OR "ALD" OR "Acral lick" OR "tail-chasing") AND ("Dogs" OR "canines")) OR (("Clomipramine" AND "fluoxetine") AND ("canine" OR "dog")) PubMed: (("clomipramine" OR "Anafranil" or "clomicalm") AND ("fluoxetine" OR "Prozac" OR "Sarafem") AND ("Anxiety" OR "behavior" OR "behaviour" OR "anxious" OR "fearful" OR "fear" OR "stress" OR "licking" OR "ALD" OR "Acral lick" OR "tail-chasing") AND ("Dogs" OR "canines")) OR (("Clomipramine" AND "fluoxetine") AND ("canine" OR "dog"))
Search terms	10 Oct 2023

**Exclusion / inclusion criteria:** 

Exclusion	Papers sampling cat populations. Papers that look at only clomipramine or fluoxetine without comparing the two. Papers that did not answer PICO question. Papers that did not specify if symptoms of anxiety were improved. Review articles. Cell models.
Inclusion	Clomipramine compared to fluoxetine. Papers written in English. Results compared in dog / canine populations. Changes in anxiety-related behaviours (tail chasing, licking, etc.).

**Search outcome:** 

Database	Number of results	Excluded – Did not use both fluoxetine and clomipramine	Excluded – Used rats or cell lines as population	Excluded – Did not answer PICO question	Excluded – Reviews	Total relevant papers
CAB Abstracts	14	1	2	1	8	2
PubMed	9	0	2	4	1	2
Total relevant papers when duplicates removed						3

### Author contributions

**Olivia Williamson:** Conceptualisation, Methodology, Investigation, Writing – Original draft, Writing – Review and Editing. **Valery Varela:** Conceptualisation, Methodology, Investigation, Writing – Original draft. **Juliana Tom:** Conceptualisation, Methodology, Investigation, Writing – Original draft, Writing – Review and Editing. **Elizabeth Powell:** Conceptualisation, Methodology, Investigation, Writing – Original draft. **Christopher Minami:** Conceptualisation, Methodology, Investigation, Writing – Original draft. **Jeffrey W. Norris:** Writing – Review and Editing, Supervision.

### ORCiD

Olivia Williamson: https://orcid.org/0000-0001-5426-080X

Valery Varela: https://orcid.org/0000-0002-1928-8193

Juliana Tom: https://orcid.org/0000-0003-2843-1219

Elizabeth Powell: https://orcid.org/0000-0001-9432-9461

Christopher Minami: https://orcid.org/0000-0002-7184-3748

Jeffrey W. Norris: https://orcid.org/0009-0009-6039-1529

### Conflict of interest

The authors declare no conflicts of interest.
